# Condoliase Injection Therapy for Lumbar Disc Herniation With Incomplete Motor Deficits in a Professional Footballer: A Case Report

**DOI:** 10.7759/cureus.79270

**Published:** 2025-02-19

**Authors:** Takeshi Seki, Takato Aihara, Kenji Endo, Yuichiro Murakami, Takahisa Haraguchi, Ryo Matsunaga, Takashi Sando, Kengo Yamamoto

**Affiliations:** 1 Orthopedic Surgery, Tokyo Medical University, Tokyo, JPN; 2 Orthopedic Surgery, Antlers Sports Clinic, Kashima, JPN

**Keywords:** athlete, condoliase, lumbar disc herniation, motor deficit, soccer player

## Abstract

Although there are reports on good short-term outcomes of condoliase injection therapy for lumbar disc herniation (LDH), reports on the improvement of motor deficits, athlete-specific outcomes, or physiotherapy are lacking. We present a case of a 20-year-old male professional footballer who had low back pain, right gluteal pain, and numbness in the right lateral lower leg. The conservative treatment for L4/L5 disc herniation did not improve his symptoms, which were accompanied by incomplete motor deficits of the tibialis anterior (TA) and extensor hallucis longus (EHL) muscles. Thus, he was administered condoliase injection therapy. Approximately 13 weeks after the injection, his symptoms had completely disappeared, and his motor deficits had improved. After 14 weeks, the patient was able to return to competition. Condoliase injection therapy for athletes is one of the useful treatments with the potential to avoid surgery and return to competition, and it may also be effective for athletes with mild incomplete motor deficits.

## Introduction

Since the initiation of Love's procedure in 1938, surgical treatment for lumbar disc herniation (LDH) has evolved toward minimally invasive surgery [1], and non-surgical treatment, such as condoliase injection therapy, which does not involve cutting into tissues or directly manipulating nerves, has been available in Japan since 2018 [2]. This treatment is considered to be somewhere between conservative treatment and surgical treatment. In cases that are resistant to conservative treatment, the responsible lesion is confirmed by nerve root block, and then condoliase injection therapy is performed. In Japan, the number of cases of condoliase injection therapy is on the rise, and it is becoming a treatment that can replace surgery [3]. In athletes, the problems with surgery for LDH include the risk of recurrence and the inability of some athletes to return to competition. Although there are reports on good short-term outcomes with condoliase injection therapy for LDH [4-6], reports summarizing improvement in motor deficits with condoliase injection therapy are lacking. There are also no reports on the progress of this therapy in athletes until they return to competition. This report describes the progress of rehabilitation and improvement of incomplete motor deficits in a professional soccer player who developed incomplete motor deficits due to LDH. He was treated with condoliase injection therapy, after which he was able to return to competition at 14 weeks.

## Case presentation

We herein present a 20-year-old male professional soccer player (midfielder) with no significant past medical history. He had been practicing for four months with right non-progressive gluteal pain. However, the pain worsened and kept him out of the game for four weeks. He was referred to our clinic with complaints of low back pain, right gluteal pain, and numbness in the right lateral lower leg. The numerical rating scale showed a high score for leg pain, and the Japanese Orthopaedic Association Back Pain Evaluation Questionnaire (JOABPEQ) [7] showed a particularly low score for social life function (Table 1).

**Table 1:** Course of NRS and JOABPEQ before and after condoliase injection therapy NRS: numerical rating scale, JOABPEQ: Japanese Orthopaedic Association Back Pain Evaluation Questionnaire

	Pre	2 weeks	3 weeks	5 weeks	9 weeks	13 weeks	27 weeks	52 weeks
NRS								
Leg pain	9	9	3	2	2	0	0	0
NRS (leg numbness)	5	5	3	2	0	0	0	0
NRS (low back pain)	2	2	3	2	1	0	0	0
JOABPEQ								
Low back pain	100	100	100	100	100	100	100	100
Lumbar function	75	75	83	100	100	100	100	100
Walking ability	43	43	93	100	100	100	100	100
Social life function	16	16	38	49	57	100	100	100
Mental health	45	78	78	66	72	86	89	89

**Figure 1:**
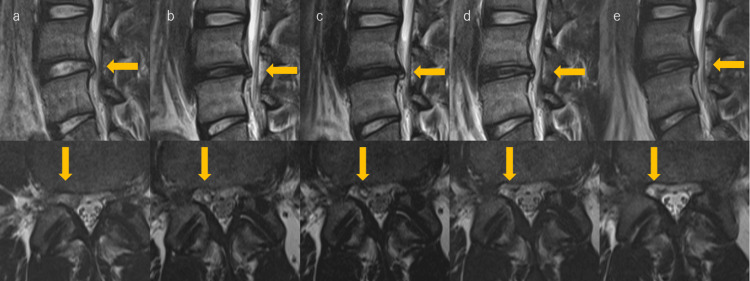
Course of L4/L5 disc herniation in the sagittal and horizontal sections of MRI (a) Preinjection, (b) four weeks after injection, (c) 13 weeks after injection, (d) 26 weeks after injection, and (e) 52 weeks after injection.

**Figure 2:**
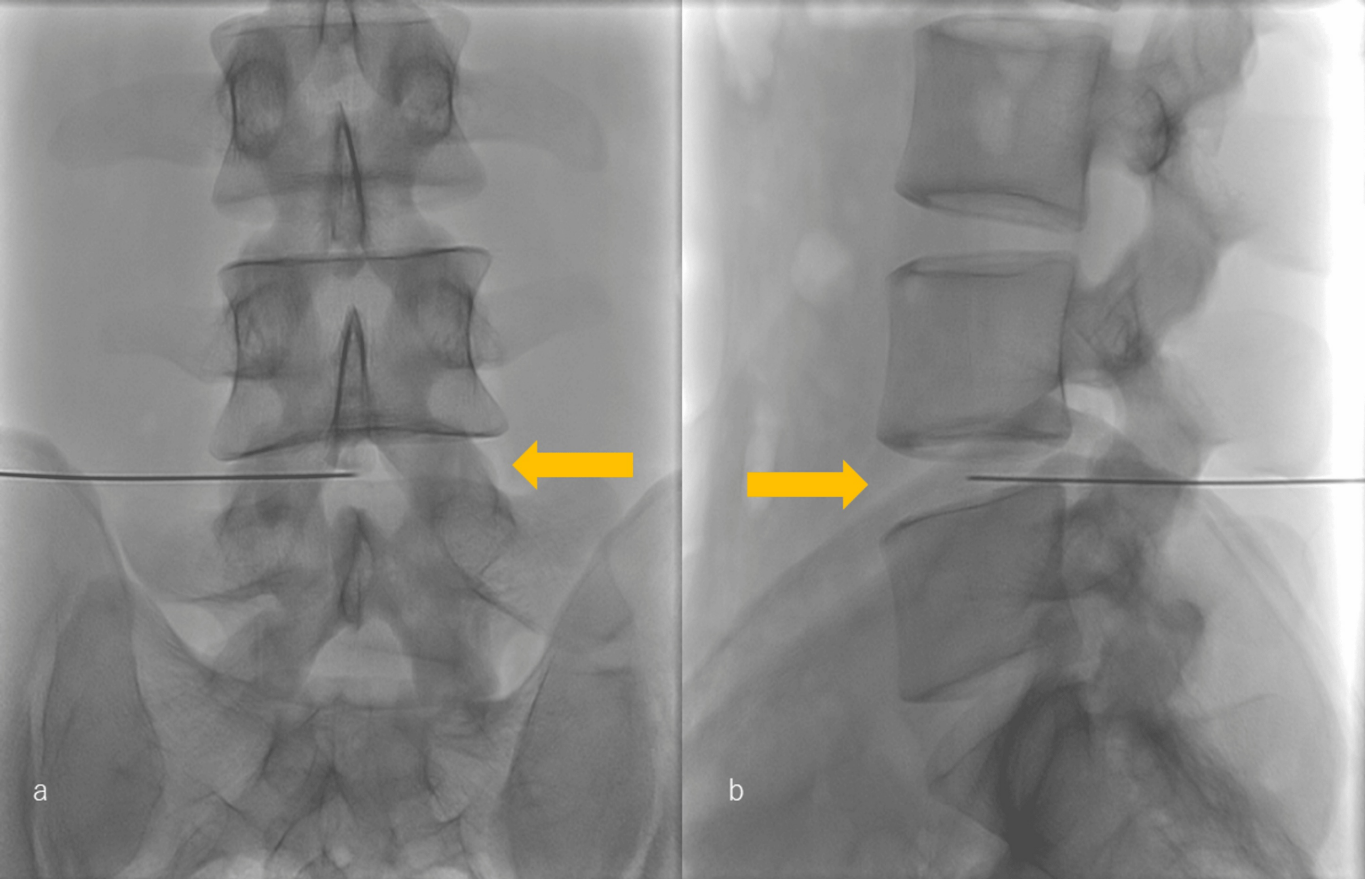
Plain radiographs of the lumbosacral spine during condoliase injection therapy The puncture needle tip is centered in both the (a) anterior-posterior and (b) lateral views.

The thoracolumbar spine was restricted in forward flexion, and the right-sided straight leg raise test was positive at 20° (Table 2). Manual muscle testing (MMT) revealed incomplete motor deficits in the right tibialis anterior (TA) and extensor hallucis longus (EHL) muscles (Table 2). MRI revealed L4/L5 disc grade III degeneration by Pfirrmann et al. [8] compressing the base of the right L5 nerve root, with a high-signal area inside the herniation on T2 images (Figure 1a). A right L5 nerve root block was performed. However, the symptoms did not improve. Therefore, condoliase injection therapy was initiated at the L4/L5 disc (Figure 2).

**Table 2:** Course of physical examination findings FFD: finger floor distance, SLRT: straight leg raise test, MMT: manual muscle testing, TA: tibialis anterior, EHL: extensor hallucis longus

	Pre	2 weeks	3 weeks	5 weeks	9 weeks	13 weeks	27 weeks	52 weeks
Forward flexion FFD (cm)	32	39	31	32	24	18	18	16
SLRT (°) (right/left)	20＋/70−	35＋/70−	30＋/70−	45＋/70−	60＋/70−	70−/70−	70−/70−	70−/70−
MMT: TA muscles (right/left)	4/5	5/5	5/5	5/5	5/5	5/5	5/5	5/5
MMT: EHL muscles (right/left)	3/5	3/5	4/5	4/5	4/5	5/5	5/5	5/5

The rehabilitation consisted of strengthening the TA and EHL muscles from the day after the condoliase injection and strength training of the quadriceps and trunk muscles from one week onward without stressing the lumbar spine. MMT revealed complete recovery of the TA muscle and partial recovery of the EHL muscle at three weeks (Table 2). After three weeks of treatment, leg pain and numbness decreased over time (Table 1). Therefore, aerobic training was started from three weeks and running and ball training from four weeks after the condoliase injection, and the exercise intensity was gradually increased. Low back pain tended to improve after nine weeks, and after 13 weeks, the symptoms completely disappeared and the patient was able to fully participate in their practices. MRI showed visualization of the right L5 nerve root four weeks after injection (Figure 1b), but a mild posterior protrusion of the disc remained at 13 weeks (Figure 1c). However, the patient had no difficulty in playing and had marked improvement in all items on the JOABPEQ at 13 weeks (Table 1). He returned to official professional games at 14 weeks and has been playing in games as a starting member at 15 weeks. One year after the injection, LDH did not recur (Figure 1e). Two years after the injection, he played the game without symptoms.

## Discussion

In many cases of surgical therapy for LDH, neurological symptoms improve immediately after surgery; to avoid LDH recurrence, rehabilitation protocols need to be planned [9]. Contrarily, with condoliase injection therapy, the drug remains in the nucleus pulposus, and the chemical changes are long-lasting, so the symptoms gradually improve [10]. Therefore, the rehabilitation protocol should be planned to account for symptom and chemical changes. Condoliase exhibits high substrate specificity for glycosaminoglycans of proteoglycans abundant in the nucleus pulposus of the intervertebral disc [11]. Glycosaminoglycans are degraded by condoliase, and the water content of proteoglycans is reduced. Thereby, the intradiscal pressure decreases, and nerve root compression is alleviated as the herniation shrinks. Variable back pain in response to chemical changes has also been observed. Chiba et al. reported that temporary back pain occurred in 36.6% of patients after condoliase injection, most of which occurred within one week after injection, and improved with follow-up [12]. Therefore, the patient in the present case was limited to TA and EHL muscle strengthening exercises only for one week after the condoliase injection. After one week, the patient began trunk exercises as well as thoracic and pelvic mobility exercises that were not stressful to the lumbar spine. Matsuyama et al. reported a maximum concentration of keratan sulfate, indicative of proteoglycan degradation, at six hours after condoliase injection and remained significantly elevated until six weeks later [10]. The keratan sulfate concentrations did not significantly increase after 13 weeks. Therefore, in the present case, rehabilitation was set up with the aim of full return to practice after 13 weeks when the chemical changes had subsided. It is difficult to create a rehabilitation protocol based solely on the concentration of keratan sulfate, so we set the rehabilitation protocol until week 13 based on the improvement of neurological symptoms and low back pain. We believe that setting a clear target of full recovery at 13 weeks after the injection is a significant advantage compared to conservative treatment, where the recovery period cannot be clearly set. It is also not significantly different from other surgical treatments.

The problem in the present case was that the patient was an elite athlete and his TA and EHL muscles had incomplete motor deficits. The return to sports and recurrence rates for each surgical procedure for LDH are summarized in Table 3.

**Table 3:** Return to sports and recurrence rates of surgical procedures for LDH LDH: lumbar disc herniation

Author	Year	Procedure	Number	Athletes or non-athletes	Return to sports rate	Return time	Recurrence	Average follow-up period
Weber et al. [13]	2009	Microdiscectomy	105	Athletes	82.9% (n=87)	5.8 months	5.7% (n=6)	-
Matsumoto et al. [14]	2013	Microendoscopic discectomy	344	Non-athletes	-	-	10.8% (n=37)	3.6 years
Chiba et al. [12]	2018	Condoliase injection therapy	82	Non-athletes	-	-	1.2% (n=1, opposite side)	52 weeks
Nakamae et al. [15]	2019	Percutaneous endoscopic discectomy	21	Athletes	95% (n=20)	9.2 weeks	5% (n=1)	28.1 months
Okada et al. [4]	2021	Condoliase injection therapy	82	Non-athletes	-	-	0%	9.1 months
Banno et al. [6]	2024	Condoliase injection therapy	67	Non-athletes	-	-	0%	2 years

With regard to the surgical outcomes for athletes, Weber et al. reported that 82.9% (87/105) of athletes returned to sports at an average of 5.8 months following microdiscectomy [13]. In the five-year follow-up, the recurrence rate was 5.7% (n = 6), with three having had herniated before returning to sporting activities. In a percutaneous endoscopic discectomy (PED), Nakamae et al. reported that 95% (20/21) of athletes were able to return to sports at a mean of 9.2 weeks postoperatively [15]. The mean postoperative follow-up periods were 28.1 months, and 5% (n = 1) of the athletes had recurrent LDH at 10 months postoperatively. However, the posterior longitudinal ligament is partially resected in any surgery; therefore, we believe that LDH recurrence is the problem, particularly in elite professional athletes. In the less-invasive microendoscopic discectomy (MED), Matsumoto et al. reported that LDH recurrence occurred in 10.8% (37/344) of non-athletes at an average of 16.6 months postoperatively as a complication following MED [14].

The advantages of condoliase injection therapy are that it is minimally invasive because it does not involve cutting into the tissue and it has a low recurrence rate because the posterior longitudinal ligament is preserved. There are no reports of condoliase injection therapy for athletes, but relatively good results have been reported in non-athletes. Okada et al. reported that 85.4% (70/82) of patients had an improvement of 50% or more in the visual analog scale of leg pain at six months following injection and that LDH did not recur after a mean follow-up period of 9.1 months [4]. Banno et al. reported no LDH recurrences at the two-year follow-up in 67 patients after injection [6]. Chiba et al. reported only one recurrence (1.2%) on the opposite side of the same elevation in 82 cases at 52 weeks following injection, which did not require surgical treatment [12]. In the present case, no LDH recurrence was observed after one year of condoliase injection therapy, and the patient played without symptoms.

For LDH with incomplete motor deficits, open surgery is often reported to ensure motor deficit decompression. However, there are few reports on endoscopic surgery (Table 4).

**Table 4:** Surgical outcomes for LDH with incomplete motor deficits LDH: lumbar disc herniation, MMT: manual muscle test, MRC: Medical Research Council

Author	Year	Procedure	Number	Degree of motor deficits	Results	Average follow-up period
Postacchini et al. [16]	2002	Microdiscectomy	116	MMT 4: n=78, MMT 3: n=24, MMT1 or 2: n=14	Complete recovery: 76% (n=88); persistent weakness was found in 16% of patients who had had a mild preoperative deficit and in 39% of those with severe or very severe weakness	6.4 years
Aono et al. [17]	2007	Standard discectomy	25	MMT ≤ 4	Complete and almost complete recovery: 52% (n=13)	3.7 years
Telfeian et al. [18]	2019	Percutaneous endoscopic discectomy	5	MMT 3: n=4, MMT 1: n=1	Complete recovery: 80% (n=4), almost complete recovery: 20% (n=1)	1 year
Kögl et al. [19]	2021	Microscopic sequestrectomy/discectomy	60	MRC > 3/5: 35, MRC ≤ 3/5: 25	Complete recovery: 58.3% (n=35), incomplete recovery: 41.7% (n=25)	1 year
Fujimoto et al. [3]	2024	Condoliase injection therapy	17	MMT 4: n=9, MMT 3: n=5, MMT 2: n=1, MMT 1: n=1, MMT 0: n=1	Recovery rates: 76%, MMT 4: 89% (8/9), MMT ≤ 3: 63% (5/8)	3 months

Aono et al. reported that in 25 patients who underwent standard discectomy for MMT 4 or less, complete and almost complete recoveries were achieved in 13 (52%) patients [17]. Kögl et al. reported on the recovery of motor deficit following lumbar microscopic sequestrectomy or discectomy in 60 patients with LDH with motor deficit [19]. The favorable prognostic factors for recovery were early surgery within 72 hours of the onset of motor deficit and mild motor deficit on the Medical Research Council scale 4. When these two conditions were met, complete recovery of motor deficit was achieved in all nine cases. Postacchini et al. performed microdiscectomy in 51 patients with MMT 4 or less in the TA muscle, of whom 40 (78%) had complete recovery of motor deficit and nine (18%) returned to almost full muscle strength [16]. In a report on endoscopic surgery, Telfeian et al. performed transforaminal discectomy of far lateral L5/S disc herniation in five patients with MMT 3 or less, with complete and almost complete recoveries of paralysis in four and one patient, respectively [18]. The transforaminal approach does not directly manipulate the posterior nerve root; therefore, there is no risk of aggravation of motor deficits due to device landing and irrigating pressure from endoscopic techniques [20].

There is only one report on the results of condoliase injection therapy in cases of incomplete motor deficits. Fujimoto et al. reported that they had performed condoliase injection therapy on 17 cases of incomplete motor paralysis, and the recovery rate was 76% [3]. The recovery rate was good at 89% for cases with an MMT of 4 or more but was 63% for cases with an MMT of 3. The fact that there is no direct manipulation of the nerve root is an advantage of condoliase injection therapy, but the fact that it takes longer to decompress the nerve than surgical treatment may be a disadvantage in terms of improving motor deficits. In this case, complete recovery was achieved even with an MMT of 3, but in order to consider the application of condoliase injection therapy to cases with an MMT of 3 or less, it will be necessary to accumulate further reports in the future. One limitation of this case is that the onset time of the incomplete motor deficits was unknown. If there are clearer reports on the relationship between the onset time of motor deficits and treatment, it will lead to a better understanding of the indications for condoliase injection therapy for LDH with incomplete motor deficits.

## Conclusions

A 20-year-old male professional soccer player who was suffering from low back pain, right gluteal pain, numbness in the right lateral part of the lower leg, and incomplete motor deficits with an MMT score of 4 for the tibialis anterior muscle and 3 for the extensor hallucis longus muscle due to L4/L5 disc herniation was treated with condoliase injection therapy. The right gluteal pain was the main cause of the decline in performance. After 13 weeks, the symptoms had completely disappeared and the motor deficits had completely improved. After 14 weeks, the patient returned to competitive play, and two years after the injection, he continued to play without pain. Condoliase injection therapy for athletes is one of the useful treatments with the potential to avoid surgery and return to competition, and it may also be effective for athletes with mild incomplete motor deficits.
